# Long-lasting effects of COVID-19 pandemic on hospitalizations and severity of bronchiolitis

**DOI:** 10.1007/s00431-023-05395-1

**Published:** 2024-01-18

**Authors:** Gregorio Paolo Milani, Andrea Ronchi, Carlo Agostoni, Paola Marchisio, Giovanna Chidini, Nicola Pesenti, Anita Bellotti, Marco Cugliari, Riccardo Crimi, Valentina Fabiano, Carlo Pietrasanta, Lorenza Pugni, Fabio Mosca, IRIDE study group, Roberta Barachetti, Claudia Pagliotta, Silvia Gulden, Francesco Maria Risso, Michael Colpani, Salvatore Aversa, Paolo Tagliabue, Federico Cattaneo, Roberta Corbetta, Maria Luisa Ventura, Stefano Ghirardello, Ilaria De Lucia, Francesca Garofoli, Luca Mancini, Giulia Angela Carla Pattarino, Costantino De Giacomo, Salvatore Barberi, Mario Vernich, Elisabetta Veronelli, Emanuela Brazzoduro, Ilaria Bottino, Tiziana Varisco, Patrizia Calzi, Alessandro Porta, Paola Alga, Laura Cozzi, Francesca Lizzoli, Lorenzo D’Antiga, Giovanna Mangili, Angelo Mazza, Fabiana Di Stasio, Gian Luigi Marseglia, Amelia Mascolo, Matea Jankovic, Lidia Decembrino, Dario Pantaleo, Chiara Vimercati, Martha Caterina Faraguna, Francesca Cattaneo, Irene Lepri, Laura Pogliani, Chiara Vimercati, Liana Bevilacqua, Luca Bernardo, Sergio Arrigoni, Giuseppe Mercurio, Costanza Paramithiotti, Elisabetta Salvatici, Giuseppe Banderali, Alberto Fabio Podestà, Elisa Dusi, Teresa Vivaldo, Sonia Bianchini, Graziano Barera, Paolo Del Barba, Claudia Aracu, Stefano Martinelli, Alice Proto, Marco Fossati, Lorella Rossi, Emilio Palumbo, Marta Odoni, Ilaria Dalla Verde, Ahmad Kantar, Paola Sindico, Grazia Morandi, Valeria Fasolato, Germana Viscogliosi, Nunzia Pia Managanelli, Giuseppe Riva, Chryssoula Tzialla, Roberta Giacchero, Caterina Sabatini, Elena Rossi, Cesare Antonio Ghitti, Ilaria Pacati, Raffaele Badolato, Laura Dotta, Antonella Meini, Ilia Bresesti, Antonio Francone, Anna Maria Plebani, Massimo Agosti, Lorella Rossi, Marco Sala, Simona Santucci, Chiara Cuzzupè, Cristina Bellan, Federica Pontiggia, Alice Romero, Chiara Perazzi, Anna Banfi, Gianvincenzo Zuccotti, Gianluca Lista

**Affiliations:** 1 Pediatric Unit, Fondazione IRCCS Ca’ Granda Ospedale Maggiore Policlinico, Milan, Italy; 2 Department of Clinical Sciences and Community Health, Università Degli Studi Di Milano, Via Della Commenda 9, 20122 Milan, Italy; 3 Neonatal Intensive Care Unit, Fondazione IRCCS Ca’ Granda Ospedale Maggiore Policlinico, Milan, Italy; 4 Department of Pathophysiology and Transplantation, University of Milan, Milan, Italy; 5 Department of Anaesthesia, Intensive Care and Emergency, Fondazione IRCCS Ca’ Granda Ospedale Maggiore Policlinico, Milan, Italy; 6 Pediatric Department, “Vittore Buzzi” Children’s Hospital, Milan, Italy; 7 Department of Biomedical and Clinical Science, University of Milan, Milan, Italy

**Keywords:** Bronchiolitis, Epidemiology, Pandemic, Risk factors, Immune debt

## Abstract

Bronchiolitis is a common cause of hospitalization in infants. The long-lasting impact of hygiene and social behavior changes during the pandemic on this disease is debated. We investigated the prevalence of hospitalized cases, clinical severity, and underlying risk factors before and during pandemic. The study was conducted in 27 hospitals in Italy and included infants hospitalized for bronchiolitis during the following four periods: July 2018-March 2019, July 2020-March 2021, July 2021-March 2022, and July 2022-March 2023. Data on demographics, neonatal gestational age, breastfeeding history, underlying chronic diseases, presence of older siblings, etiologic agents, clinical course and outcome were collected. A total of 5330 patients were included in the study. Compared to 2018–19 (n = 1618), the number of hospitalizations decreased in 2020–21 (n = 121). A gradual increase was observed in 2021–22 (n = 1577) and 2022–23 (n = 2014). A higher disease severity (need and length of O_2_-supplementation, need for non-invasive ventilation, hospital stay) occurred in the 2021–22 and, especially, the 2022–23 periods compared to 2018–19. This tendency persisted after adjusting for risk factors associated with bronchiolitis severity.

*   Conclusions*: Compared to adults, COVID-19 in infants is often asymptomatic or mildly symptomatic and rarely results in hospitalization. This study indicates that the pandemic has indirectly induced an increased burden of bronchiolitis among hospitalized infants. This shift, which is not explained by the recognized risk factors, suggests the existence of higher infant vulnerability during the last two seasons.

**Table:** 

**What is known:** *• The pandemic led to a change in epidemiology of respiratory diseases* *• Large data on severity of bronchiolitis and underlying risk factors before and during COVID-19 pandemic are scarce*	
**What is new:** *• Compared to pre-pandemic period, hospitalizations for bronchiolitis decreased in 2020–21 and gradually increased in 2021–22 and 2022–23* *• Compared to pre-pandemic period, higher disease burden occurred in 2021–22 and, especially, in 2022–23. This tendency persisted after adjusting for risk factors associated with bronchiolitis severity* *• The interplay among viruses, preventive measures, and the infant health deserves to be further investigated*

**Supplementary Information:**

The online version contains supplementary material available at 10.1007/s00431-023-05395-1.

## Introduction

Bronchiolitis, a lower respiratory tract infection affecting the small airways, is a common cause of hospitalization in infancy [1]. In the Northern Hemisphere, bronchiolitis typically occurs from October to March and is a recognized burden for hospital services [2–4]. A drastic reduction in bronchiolitis cases was observed during the first pandemic year (2020–21) [5, 6]. Social distancing, reduction of day care attendance, hygienic practices such as handwashing with antiseptic gels, and the use of protective masks by adults and older children are believed to be responsible for this shift [7]. During the second year of the pandemic (2021–22), an increased number of bronchiolitis with more severe cases often occurring before the autumn-winter season has been observed [8, 9]. Some authors hypothesized that the limited exposure to microorganisms during the first pandemic year led to a higher number of severe cases than suggested by historical data [8]. However, other factors that emerged during the pandemic have also been claimed to favor this shift. First, a drastic change in respiratory syncytial virus epidemiology, the main cause of bronchiolitis [10, 11]. Second, a decrease in breastfeeding practice, a protective factor against severe bronchiolitis [12–14]. Finally, older siblings, who had not been exposed to respiratory viruses during the initial pandemic phase, might have facilitated an increased viral transmission after the relaxation of preventive measures in the following years [15, 16].

Limited epidemiological data support these hypotheses [10]. Furthermore, little evidence is available on bronchiolitis during the season 2022–23. We conducted a multicenter study on infants hospitalized for bronchiolitis in the Lombardy region (Italy), which is considered the first COVID-19 epicenter in Europe. The primary aim of this study was to assess the prevalence and clinical severity of hospitalized cases before and during the pandemic. The secondary aim was to test whether main known risk factors explain the possible changes in bronchiolitis severity.

## Methods

The IRIDE (“Investigating bRonchiolitis epidemiology During the pandemic Emergency”) study is a multicenter observational cohort study conducted in 27 hospitals in Lombardy, Italy. These hospitals, whose list is given in the supplementary online material (Table S1), account for > 80% of hospital bed capacity for pediatric patients in Lombardy. Eligible for the study were infants aged < 24 months, hospitalized for at least 12 h with a diagnosis of bronchiolitis and discharged by one of the hospitals participating in the study during the following four periods: July 2018—March 2019 (pre-pandemic period), July 2020—March 2021, July 2021—March 2022, and July 2022—March 2023. Since the outbreak of SARS-CoV-2 and the first restrictive measures to limit its spread occurred in Lombardy at the beginning of 2020, the period 2019–2020 was not considered. On the other hand, the three pandemic periods considered in this study were characterized by a gradual decrease in preventive and hygiene measures against viral circulation [16, 17]. Bronchiolitis cases not requiring hospitalization were excluded. To prevent patients transferred from one hospital to another from being enrolled twice, each center could recruit only those patients who had been discharged from their own facility.

**Table 1:** Demographics, clinical and microbiological characteristics of included infants in the four study periods

	**2018–2019**	**2020–2021**	**2021–2022**	**2022–2023**	**p**
N	1618	121	1577	2014	
Age, months	3.0 [1.0–5.0]	5.6 [1.5–9.0]	2.0 [1.0–4.3]	2.5 [1.0–5.5]	< 0.001
Age > 12 months	96 (5.9)	23 (19)	77 (4.9)	140 (7.0)	< 0.001
Sex					
Female	683 (42)	36 (30)	707 (45)	849 (42)	0.009
Male	935 (58)	85 (70)	870 (55)	1165 (58)	
Cases before autumn–winter season^a^	24 (1.5)	25 (20)	25 (1.6)	38 (1.9)	< 0.001
Number of older siblings	1 [1-1]	1 [0–1]	1 [1-1]	1 [1-1]	0.012
Maternal COVID-19 during pregnancy	0 (0.0)	2 (2.3)	20 (1.8)	63 (5.8)	< 0.001
Neonatal gestational age, weeks	38.2 (± 2.7)	37.6 (± 3.2)	38.2 (± 2.7)	38.2 (± 2.6)	0.124
Neonatal body weight, grams	3101 (± 656)	2957 (± 779)	3137 (± 657)	3117 (± 635)	0.043
Breastfeeding	970 (76)	64 (69)	1040 (76)	1261 (78)	0.099
Length of exclusive breastfeeding, months	2.0 [1.0–4.0]	1.5 [0.0–5.0]	1.5 [1.0–3.1]	2.0 [1.0–4.0]	0.057
Chronic disease	105 (6.5)	16 (13)	126 (8.0)	124 (6.2)	0.006
Bronchopulmonary dysplasia	20 (1.2)	5 (4.1)	21 (1.3)	25 (1.2)	0.059
Congenital heart disease	11 (0.7)	2 (1.7)	27 (1.7)	19 (0.9)	0.032
Further	74 (4.6)	9 (7.4)	78 (4.9)	80 (4.0)	0.201
Radiologically confirmed pneumonia					
Yes	292 (18)	14 (12)	277 (18)	322 (16)	0.152
No	509 (32)	50 (41)	495 (31)	480 (24)	
Rx not performed	814 (50)	57 (47)	805 (51)	1180 (60)	
Etiologic agent					
Respiratory syncytial virus	897 (56)	8 (6.6)	1120 (71)	1267 (63)	< 0.001
Other infectious agents	271 (17)	63 (52)	184 (12)	321 (16)	
No agent identified	450 (28)	50 (41)	273 (17)	426 (21)	

Data are presented as median [IQR], mean (± SD) or absolute frequency (percentage). ANOVA or Kruskal-Wallis test for continuous variables, and Fisher exact test or Chi-squared test for categorical variables were used for comparison

^a^July–September

The following ICD-9 codes were initially used: 46,611 (respiratory syncytial virus bronchiolitis), 46,619 (bronchiolitis caused by other infectious agents), 4801 (pneumonia due to respiratory syncytial virus), 0796 (respiratory syncytial virus infection), 51,881 (respiratory failure in children), 77,084 and 77,089 (respiratory failure in neonates). Subsequently, the clinical charts were retrieved, and only subjects with a history of acute respiratory tract infection of the upper airways in the previous days, followed by an acute onset of cough, diffuse crackles, and respiratory distress, were retained [4, 18]. For included subjects, the following data were retrospectively extracted from the clinical records: age at admission, sex, neonatal gestational age and body weight, any breastfeeding and duration of exclusive breastfeeding, number of older siblings, underlying chronic diseases that predispose to severe bronchiolitis (including bronchopulmonary dysplasia and severe congenital heart disease), occurrence of radiologically confirmed diagnosis of pneumonia during the bronchiolitis episode, infectious agent detected by nasopharyngeal aspirate, need and duration of O_2_-supplementation, use of non-invasive ventilation support (including Bi-PAP and C-PAP), need and duration of intensive care, need for invasive ventilation support, use of extracorporeal membrane oxygenation (ECMO), length of the whole hospital stay. Furthermore, fatal cases were also considered. All data were gathered and de-identified at each study center. They were then collected using REDCap tools hosted at the Ospedale Maggiore, Policlinico, Milan, Italy. The study was approved by the Ethical Committee of the coordinating center (Comitato Etico Milano Area2, Milan, code 186796, date of approval April 26, 2023) and by the other participating centers.

### Statistical analysis

The distribution of continuous variables was visually tested using histograms and density plots. Parametric data were presented as mean and standard deviations and non-parametric data as median and interquartile range [IQR]. Categorical data were presented as absolute and relative frequencies. ANOVA and Kruskal-Wallis test were used to compare continuous variables, and Fisher exact test or Chi-squared test were used to compare categorical variables, as appropriate. The Bonferroni correction was used for multiple comparisons.

Mixed effect regression models were used to study differences in outcomes between the study periods, with the hospital as a random effect. Models’ results are expressed as linear coefficients or odds ratios for continuous and categorical outcomes, respectively, with 95% CI and p-values. Categorical outcomes included the need for O_2_-supplementation, use of non-invasive or invasive ventilation and the need for intensive care. Continuous outcomes included the length of O_2_-supplementation, intensive care stay and overall hospitalization. The four study periods (2018–19, 2020–21, 2021–22 and 2022–23) were tested as predictive variable. All models were adjusted for typical (age, sex, gestational age at birth, underlying chronic disease and positive testing for respiratory syncytial virus) and recently recognized (history of breastfeeding and number of older siblings) confounders of bronchiolitis severity. The models were performed both including all patients and then including only patients 12 months of age or less. Statistical significance was set at p < 0.05. The R software (R Foundation for Statistical Computing, Vienna, Austria) was used for the analysis.

## Results

A total of 5330 patients hospitalized for bronchiolitis were included in the study. The median age of the included subjects was 2.3 [IQR 1.0–5.0] months, and 57% were males. The mean neonatal gestational age was 38.2 (± 2.7) weeks, and 957 (76%) had a history of breastfeeding. A total of 2479 (47%) patients had at least one older sibling, while 371 (6.9%) had an underlying chronic disease. A pneumonia was diagnosed in 905 (17%) infants. Respiratory syncytial virus was detected in 3292 (62%) cases. During hospitalization, 3712 (70%) patients required O_2_-supplementation, 2293 (43%) received non-invasive ventilation support, 78 (1.6%) required invasive ventilation support, and 6 (0.1%) were treated with ECMO. Three patients died in 2018–19 and one in 2021–22.

Data divided for the four study periods are reported in Table 1. The results of comparison between 2018–19 and the other periods are detailed in the online Supplementary Material.

Considering the main outcomes of the analysis, differences among the four periods were observed for the need and length of O_2_-supplementation, the need for and length of intensive care, and the length of the whole hospitalization. No difference was observed in the need for invasive ventilation support or ECMO (Table 2).

**Table 2:** Bronchiolitis course and outcome in the four different study periodsData are presented as median [IQR] or absolute frequency (percentage). ANOVA or Kruskal-Wallis test for continuous variables, and Fisher exact test or Chi-squared test for categorical variables were used for comparison

	**2018–2019**	**2020–2021**	**2021–2022**	**2022–2023**	**p**
N	1618	121	1577	2014	
Oxygen supplementation	959 (59)	69 (57)	1157 (73)	1527 (76)	< 0.001
Length of oxygen supplementation, days	0 [0–4]	1 [0–3]	2 [0–5]	2 [0–5]	< 0.001
Non-invasive ventilation support	470 (29)	42 (35)	726 (46)	1055 (52)	< 0.001
Invasive ventilation support	20 (1.2)	1 (0.8)	18 (1.1)	39 (1.9)	0.159
Intensive care unit admission	178 (11)	13 (11)	239 (15)	320 (16)	< 0.001
Length of intensive care stay, days	5 [3-7]	3 [2-6]	5 [3-7]	5 [4-8]	< 0.001
Length of intensive care stay ≥ 3, days	143 (80)	8 (62)	192 (80)	285 (89)	0.001
Extracorporeal membrane oxygenation	3 (0.2)	0 (0.0)	1 (0.1)	2 (0.1)	0.735
Length of the whole hospitalization, days	6 [4-8]	5 [4-6]	6 [4-8]	6 [4-8]	< 0.001
Length of the whole hospitalization ≥ 4 days	1352 (84)	91 (75)	1343 (85)	1758 (87)	< 0.001

Data are presented as median [IQR] or absolute frequency (percentage). ANOVA or Kruskal-Wallis test for continuous variables, and Fisher exact test or Chi-squared test for categorical variables were used for comparison

The results of the multiple models investigating the relation between bronchiolitis course and the pandemic periods after adjusting for typical and more recently recognized confounders are reported in Tables 3 and 4, respectively. The periods 2021–22 and 2022–23 were significantly associated with a higher need and length of O_2_-supplementation, increased use of non-invasive ventilation, and a longer hospitalization. No association among periods and the use of invasive ventilation support, and the need or length of intensive care was observed. The results of the models including exclusively patients 12 months of age or less (n = 4914) provided similar results (data are given in the online supplementary material, Tables S2 and S3).

**Table 3:** Results of the mixed effect regression models: oxygen supplementation, non-invasive ventilation support, invasive ventilation support, intensive care admission were the dependent variables. Study periods (reference 2018–19) were the predictive variables

**Outcome**	**Study periods**	**Odds ratio**	**Lower 95% confidence interval**	**Upper 95% confidence interval**	**p**
Need for oxygen supplementation	2020–21	0.701	0.390	1.258	0.233
	2021–22	1.649	1.289	2.109	< 0.001
	2022–23	2.205	1.685	2.885	< 0.001
Need for non-invasive ventilation support	2020–21	1.642	0.858	3.145	0.134
	2021–22	2.185	1.687	2.831	< 0.001
	2022–23	3.643	2.752	4.824	< 0.001
Need for invasive ventilation support	2020–21	2.175	0.241	19.618	0.489
	2021–22	0.592	0.225	1.555	0.287
	2022–23	1.514	0.651	3.523	0.336
Need for intensive care unit admission	2020–21	1.461	0.565	3.781	0.434
	2021–22	1.331	0.931	1.903	0.117
	2022–23	1.306	0.883	1.931	0.181

Models were adjusted for age, sex, gestational age at birth, underlying chronic disease (yes vs no), history of breastfeeding, number of older siblings (no older sibling vs one or more older siblings) and testing positive for respiratory syncytial virus

**Table 4:** Results of the mixed effect regression models: length of intensive care unit stay, oxygen supplementation, and overall hospitalization were the dependent variables. Study periods (reference 2018–19) were the predictive variables

**Outcome**	**Study periods**	**ß**	**Lower 95% confidence interval**	**Upper 95% confidence interval**	**p**
Length of intensive care unit stay	2020–21	0.194	-0.468	0.856	0.565
	2021–22	0.035	-0.224	0.293	0.793
	2022–23	0.101	-0.175	0.377	0.474
Length of oxygen supplementation	2020–21	-0.637	-1.429	0.155	0.115
	2021–22	0.750	0.441	1.059	< 0.001
	2022–23	1.121	0.793	1.450	< 0.001
Whole duration of hospital stay	2020–21	-0.110	-1.068	0.849	0.822
	2021–22	-0.060	-0.440	0.319	0.754
	2022–23	0.637	0.233	1.042	0.002

Models were adjusted for age, sex, gestational age at birth, underlying chronic disease (yes vs no), history of breastfeeding, number of older siblings (no older sibling vs one or more older siblings) and testing positive for respiratory syncytial virus

## Discussion

The IRIDE study investigated the epidemiology and clinical course of bronchiolitis in hospitalized infants during the pre-pandemic and pandemic periods. The study reveals a relevant reduction in bronchiolitis cases during the season 2020–21. More interestingly, it shows an increase in the number of hospitalizations, in the need and length of O_2_-supplementation, in the use of non-invasive ventilation, and a longer hospital stay in 2021–22 and, especially, in 2022–23 as compared to the pre-pandemic season. This tendency persists after adjusting for both typical risk factors for severe bronchiolitis such as young age, male sex, low neonatal gestational age, underlying chronic disease and the positivity for respiratory syncytial virus, and more recently recognized risk factors such as a decrease in breastfeeding and the presence of older siblings. Moreover, the number of patients requiring intensive care increased in 2021–22 and 2022–23. Nonetheless, the number of patients requiring invasive ventilation support and the length of intensive care stay did not change.

The drastic reduction in bronchiolitis during the season 2020–21 has been described in previous studies and confirms the efficacy of various preventive measures, such as social distancing, reduction of day care attendance, handwashing, and the use of masks [19, 20]. After the relaxation of pandemic-related measures, there was a significant increase in bronchiolitis burden in 2021–22 and 2022–23. Our data are supported by the literature, which confirms that the typical risk factors for severe bronchiolitis do not account for the observed tendency [13, 21, 22]. Decrease in breastfeeding and the presence of older siblings might also be new potential modulators of bronchiolitis severity [13, 21]. This comprehensive large-scale study shows for the first time that neither the main typical nor the newly recognized risk factors fully explain the higher burden of bronchiolitis observed in the pediatric wards during 2021–22 and 2022–23. It might be argued that the increased percentage of infants requiring O_2_-supplementation or non-invasive ventilation depends on a change in clinical practice over time (e.g., a higher threshold for hospitalization and a higher availability of devices for ventilation, respectively). However, the dramatic increase in the absolute number of infants requiring O_2_-supplementation from 2018–19 to 2012–22 (+ 21%) and 2022–23 (+ 60%) and of infants requiring intensive care (+ 54% and + 80%, respectively) does not support the latter hypothesis. Furthermore, recommendations for hospitalization and management did not vary during the study period in Italy [23]. This unexplained greater impact of bronchiolitis compared to the pre-pandemic period suggests the existence of higher vulnerability among infants. Notably, this vulnerability appears to persist for at least two bronchiolitis seasons. This hypothesis is in line with the findings of another study with a similar sample size including children affected with RSV infection in Spain [24].

The bronchiolitis burden was stronger in 2022–23 than in 2021–22. We have no clear-cut explanation for this finding. It is tempting to assume that a further relaxation of restrictive measures in 2022–23 accounts, at least in part, for this observation.

The number of patients requiring intensive care increased in 2021–22 and in 2022–23 compared to that of the pre-pandemic period. Nonetheless, the number of patients requiring invasive ventilation support and the length of intensive care stay remained stable. The results of the regression analysis suggest that low neonatal gestational age and underlying chronic diseases played a crucial role in the increased need for intensive care [25, 26].

This study has some limitations. It relied on retrospective design. ICD-10 codes were not used as they are not yet implemented for discharge diagnosis coding in Italy. Additionally, the use of ICD codes for case identification might not have captured all cases accurately. To counterbalance this risk, we used a wide range of codes and, then, all diagnoses were reviewed according to well-established criteria. Furthermore, our analysis focused uniquely on hospitalized infants. This decision was made because during the first two pandemic years, many infants with mild diseases that were previously managed uniquely by primary care physicians were directed to the emergency units. This redirection was partly due to organizational challenges in providing care to infants with a potential SARS-CoV-2 infection outside the hospital setting [27, 28]. Therefore, including infants who were not hospitalized might overestimate cases requiring emergency care during the first pandemic phases. The management of bronchiolitis may differ among centers. Mixed effects models were employed to reduce the potential for this issue. The study strengths lie in its large sample size, the high number of involved centers, and the collection of individual clinical and laboratory data. Furthermore, the main risk factors for severe bronchiolitis were considered. Finally, we did not have data on the immunological profile (e.g., levels of immunoglobulins) or changes in the respiratory microbiome over the four study periods. Therefore, the hypothesis of higher vulnerability remains only speculative.

## Conclusions

Compared to adults, COVID-19 in infants is often asymptomatic or mildly symptomatic and rarely results in hospitalization [28, 29]. The IRIDE study indicates that the pandemic has indirectly induced an increased prevalence and severity of bronchiolitis requiring hospitalization. This shift, which is not explained by the main typical or newly recognized risk factors for severe bronchiolitis, suggests the existence of higher infant vulnerability during the last two seasons.

### Supplementary Information

Below is the link to the electronic supplementary material.

Supplementary file1 (DOCX 25.4 KB)

## Data Availability

Upon reasonable request to the corresponding author.

## List of abbreviations

COVID-19: Coronavirus Disease 2019
ECMO: Extracorporeal Membrane Oxygenation
