# Correction to: Long-lasting effects of COVID-19 pandemic on hospitalizations and severity of bronchiolitis

**DOI:** 10.1007/s00431-024-05707-z

**Published:** 2024-08-07

**Authors:** Gregorio Paolo Milani, Andrea Ronchi, Carlo Agostoni, Paola Marchisio, Giovanna Chidini, Nicola Pesenti, Anita Bellotti, Marco Cugliari, Riccardo Crimi, Valentina Fabiano, Carlo Pietrasanta, Lorenza Pugni, Fabio Mosca, IRIDE study group

**Affiliations:** 1 Pediatric Unit, Fondazione IRCCS Ca’ Granda Ospedale Maggiore Policlinico, Milan, Italy; 2 Department of Clinical Sciences and Community Health, Università Degli Studi Di Milano, Via Della Commenda 9, 20122 Milan, Italy; 3 Neonatal Intensive Care Unit, Fondazione IRCCS Ca’ Granda Ospedale Maggiore Policlinico, Milan, Italy; 4 Department of Pathophysiology and Transplantation, University of Milan, Milan, Italy; 5 Department of Anaesthesia, Intensive Care and Emergency, Fondazione IRCCS Ca’ Granda Ospedale Maggiore Policlinico, Milan, Italy; 6 Pediatric Department, Vittore Buzzi” Children’s Hospital, Milan, Italy; 7 Department of Biomedical and Clinical Science, University of Milan, Milan, Italy

**Correction to: European Journal of Pediatrics (2024) 183:1751-1758**

10.1007/s00431-023-05395-1

Unfortunately, the original published online version of this article entitled "Long-lasting effects of COVID-19 pandemic on hospitalizations and severity of bronchiolitis" contained a mistake. Some of the list of authors under the IRIDE study group are missing. The complete list of authors and their corresponding affiliations of the IRIDE study group is shown below:

**IRIDE study group** Roberta Barachetti, Ospedale Sant’Anna di Como, Como, Italy; Claudia Pagliotta, Ospedale Sant’Anna di Como, Como, Italy; Silvia Gulden, Ospedale Sant’Anna di Como, Como, Italy; Francesco Maria Risso, Neonatal intensive care unit, Ospedali Civili di Brescia, Brescia, Italy; Michael Colpani, Neonatal intensive care unit, Ospedali Civili di Brescia, Brescia, Italy; Salvatore Aversa, Neonatal intensive care unit, Ospedali Civili di Brescia, Brescia, Italy; Paolo Tagliabue, S.C. Neonatologia e Terapia Intensiva Neonatale, IRCCS Fondazione San Gerardo dei Tintori, Monza, Italy; Federico Cattaneo, S.C. Neonatologia e Terapia Intensiva Neonatale, IRCCS Fondazione San Gerardo dei Tintori, Monza, Italy; Roberta Corbetta, S.C. Neonatologia e Terapia Intensiva Neonatale, IRCCS Fondazione San Gerardo dei Tintori, Monza, Italy; Maria Luisa Ventura, S.C. Neonatologia e Terapia Intensiva Neonatale, IRCCS Fondazione San Gerardo dei Tintori, Monza, Italy; Stefano Ghirardello, S.C. Neonatologia e Terapia Intensiva Neonatale, Fondazione IRCCS Policlinico San Matteo, Pavia, Italy; Ilaria De Lucia, S.C. Neonatologia e Terapia Intensiva Neonatale, Fondazione IRCCS Policlinico San Matteo, Pavia, Italy; Francesca Garofoli, S.C. Neonatologia e Terapia Intensiva Neonatale, Fondazione IRCCS Policlinico San Matteo, Pavia, Italy; Luca Mancini, Pediatria, Asst Grande Ospedale Metropolitano Niguarda di Milano, Milan, Italy; Giulia Angela Carla Pattarino, Pediatria, Asst Grande Ospedale Metropolitano Niguarda di Milano, Milan, Italy; Costantino De Giacomo, Pediatria, Asst Grande Ospedale Metropolitano Niguarda di Milano, Milan, Italy; Salvatore Barberi, ASST Rhodense Rho and Garbagnate Milanese, Italy; Mario Vernich, ASST Rhodense Rho and Garbagnate Milanese, Italy; Elisabetta Veronelli, ASST Rhodense Rho and Garbagnate Milanese, Italy; Emanuela Brazzoduro, ASST Rhodense Rho and Garbagnate Milanese, Italy; Ilaria Bottino, ASST Brianza - Ospedale Pio XI Desio, Italy; Tiziana Varisco, ASST Brianza - Ospedale Pio XI Desio, Italy; Patrizia Calzi, ASST Brianza - Ospedale Pio XI Desio, Italy; Alessandro Porta, ASST Ovest Milanese, Ospedale Fornaroli di Magenta, Italy; Paola Alga, ASST Ovest Milanese, Ospedale Fornaroli di Magenta, Italy; Laura Cozzi, ASST Ovest Milanese, Ospedale Fornaroli di Magenta, Italy; Francesca Lizzoli, ASST Ovest Milanese, Ospedale Fornaroli di Magenta, Italy; Lorenzo D'Antiga, SS Pediatria Internistica, SC Pediatria generale, ASST Papa Giovanni XXIII, Bergamo, Italy; Angelo Mazza, SS Pediatria Internistica, SC Pediatria generale, ASST Papa Giovanni XXIII, Bergamo, Italy; Fabiana Di Stasio, SS Pediatria Internistica, SC Pediatria generale, ASST Papa Giovanni XXIII, Bergamo, Italy; Gian Luigi Marseglia, Pediatria, Fondazione IRCCS Policlinico San Matteo, Pavia, Italy, Italy; Mascolo Amelia, Pediatria, Fondazione IRCCS Policlinico San Matteo, Pavia, Italy, Italy; Matea Jankovic, Pediatria, Fondazione IRCCS Policlinico San Matteo, Pavia, Italy; Lidia Decembrino, ASST Pavia, Ospedale Civile di Vigevano, Italy; Dario Pantaleo, ASST Pavia, Ospedale Civile di Vigevano, Italy; Chiara Vimercati, UO Pediatria, Ospedale San Gerardo Di Monza, Italy; Martha Caterina Faraguna, UO Pediatria, Ospedale San Gerardo Di Monza, Italy; Francesca Cattaneo, UO Pediatria, Ospedale San Gerardo Di Monza, Italy; Irene Lepri, UO Pediatria, Ospedale San Gerardo Di Monza, Italy; Laura Pogliani, ASST OVEST MI Ospedale di Legnano; Liana Bevilacqua, ASST OVEST MI Ospedale di Legnano; Luca Bernardo, ASST FateBeneFratelli Macedonio Melloni - Presidio Ospedaliero Macedonio Melloni di Milano, Italy; Sergio Arrigoni, ASST FateBeneFratelli Macedonio Melloni - Presidio Ospedaliero Macedonio Melloni di Milano, Italy; Giuseppe Mercurio, ASST FateBeneFratelli Macedonio Melloni - Presidio Ospedaliero Macedonio Melloni di Milano, Italy; Costanza Paramithiotti, SC Pediatria, Presidio San Paolo, ASST Santipaolocarlo, Milan, Italy; Elisabetta Salvatici, SC Pediatria, Presidio San Paolo, ASST Santipaolocarlo, Milan, Italy; Giuseppe Banderali, SC Pediatria, Presidio San Paolo, ASST Santipaolocarlo, Milan, Italy; Alberto Fabio Podestà, ASST Santi Paolo Carlo, Ospedale San Carlo di Milano, Milan, Italy; Elisa Dusi, ASST Santi Paolo Carlo, Ospedale San Carlo di Milano, Milan, Italy; Teresa Vivaldo, ASST Santi Paolo Carlo, Ospedale San Carlo di Milano, Milan, Italy; Sonia Bianchini, ASST Santi Paolo Carlo, Ospedale San Carlo di Milano, Milan, Italy; Paolo Del Barba, IRCCS Ospedale San Raffaele, Milan, Italy; Graziano Barera, IRCCS Ospedale San Raffaele, Milan, Italy; Claudia Aracu, IRCCS Ospedale San Raffaele, Milan, Italy; Stefano Martinelli, S.C. Neonatologia e Terapia Intensiva Neonatale, ASST Grande Ospedale Metropolitano Niguarda di Milano, Milan, Italy; Alice Proto, S.C. Neonatologia e Terapia Intensiva Neonatale, ASST Grande Ospedale Metropolitano Niguarda di Milano, Milan, Italy; Marco Fossati, S.C. Neonatologia e Terapia Intensiva Neonatale, ASST Grande Ospedale Metropolitano Niguarda di Milano, Milan, Italy; Lorella Rossi, ASST Valtellina-Alto Lario, Presidio Ospedaliero Di Sondrio, Italy; Emilio Palumbo, ASST Valtellina-Alto Lario, Presidio Ospedaliero Di Sondrio, Italy; Marta Odoni, Gruppo San Donato, Policlinico Ponte San Pietro di San Pietro, Italy; Ilaria Dalla Verde, Gruppo San Donato, Policlinico Ponte San Pietro di San Pietro, Italy; Ahmad Kantar, Gruppo San Donato, Policlinico Ponte San Pietro di San Pietro, Italy; Paola Sindico, ASST Mantova, A. O. Istituti Ospitalieri Carlo Poma di Mantova, Mantua, Italy; Grazia Morandi, ASST Mantova, A. O. Istituti Ospitalieri Carlo Poma di Mantova, Mantua, Italy; Valeria Fasolato, ASST Mantova, A. O. Istituti Ospitalieri Carlo Poma di Mantova, Mantua, Italy; Germana Viscogliosi, Pediatria, Fondazione Poliambulanza Istituto Ospedaliero di Brescia, Italy; Nunzia Managanelli, Pediatria, Fondazione Poliambulanza Istituto Ospedaliero di Brescia, Italy; Giuseppe Riva, Pediatria, Fondazione Poliambulanza Istituto Ospedaliero di Brescia, Italy; Chryssoula Tzialla, ASST Pavia - Ospedale Civile di Voghera, Italy; Roberta Giacchero, ASST Lodi, Ospedale Maggiore di Lodi, Italy; Caterina Sabatini, ASST Lodi, Ospedale Maggiore di Lodi, Italy; Elena Rossi, ASST Lodi, Ospedale Maggiore di Lodi, Italy; Cesare Antonio Ghitti, ASST Bergamo Est - Ospedale Pesenti Fenaroli Di Alzano Lombardo, Italy; Ilaria Pacati, ASST Bergamo Est - Ospedale Pesenti Fenaroli Di Alzano Lombardo, Italy; Raffaele Badolato, ASST Degli Spedali Civili di Brescia, Brescia, Italy; Laura Dotta, ASST Degli Spedali Civili di Brescia, Brescia, Italy; Antonella Meini, ASST Degli Spedali Civili di Brescia, Brescia, Italy; Ilia Bresesti, ASST Settelaghi, Ospedale “F. Del Ponte”, Varese, Italy; Antonio Francone, ASST Settelaghi, Ospedale “F. Del Ponte”, Varese, Italy; Anna Maria Plebani, ASST Settelaghi, Ospedale “F. Del Ponte”, Varese, Italy; Massimo Agosti, ASST Settelaghi, Ospedale “F. Del Ponte”, Varese, Italy; Marco Sala, ASST della Brianza, Ospedale di Vimercate, Italy; Simona Santucci, ASST della Brianza, Ospedale di Vimercate, Italy; Chiara Cuzzupè, ASST della Brianza, Ospedale di Vimercate, Italy; Cristina Bellan, Terapia Intensiva Neonatale - ASST Bergamo Est Ospedale Bolognini Seriate Italy; Federica Pontiggia, Terapia Intensiva Neonatale - ASST Bergamo Est Ospedale Bolognini Seriate Italy;Alice Romero, Pediatric Department, "Vittore Buzzi" Children's Hospital, Università degli Studi di Milano, Milan, Italy; Chiara Perazzi, Pediatric Department, "Vittore Buzzi" Children's Hospital, Università degli Studi di Milano, Milan, Italy; Anna Banfi, Pediatric Department, "Vittore Buzzi" Children's Hospital, Università degli Studi di Milano, Milan, Italy; Gianvincenzo Zuccotti, Pediatric Department, "Vittore Buzzi" Children's Hospital, Milan, Italy, Department of Biomedical and Clinical Sciences, Università degli Studi di Milano, Milan, Italy; Gianluca Lista, Division of Neonatology, "Vittore Buzzi" Children's Hospital, Milan, Italy

The original article has been corrected.

