# Supplementary material for: Long-lasting effects of COVID-19 pandemic on hospitalizations and severity of bronchiolitis
Source: Eur J Pediatr. 2024 Jan 18;183(4):1751–8. doi: 10.1007/s00431-023-05395-1 (PMC11001736; doi:10.1007/s00431-023-05395-1)
Supplement: Supplementary file 1 — Supplementary file1 (DOCX 25.4 KB) [file 431_2023_5395_MOESM1_ESM.docx]

**Supplementary online material**

**Long-lasting effects of the COVID-19 pandemic on hospitalizations and severity of bronchiolitis**

**Table S1.** List of the participating hospitals and number of enrolled subjects per each hospital.

| **Center** | **N** |
| --- | --- |
| **Total** | **5330** |
| ASST Lodi | 222 |
| ASST Papa Giovanni XXIII Bergamo | 85 |
| ASST Pavia | 191 |
| Ospedale di Rho | 135 |
| ASST Santi Paolo e Carlo Milano | 176 |
| ASST Bergamo est | 46 |
| Fondazione Poliambulanze Brescia | 435 |
| Ospedale di Garbagnate Milanese | 184 |
| Ospedale San Gerardo dei Tintori Monza e Brianza | 198 |
| Ospedale Macedonio Melloni Milano | 239 |
| ASST Mantova | 61 |
| Ospedale Niguarda Milano | 247 |
| Ospedale Bolognini Seriate | 150 |
| Ospedale Civile Voghera | 89 |
| Ospedale Pio XI Desio | 145 |
| Ospedale G. Fornaroli Magenta | 262 |
| Ospedale San Paolo Milano | 160 |
| Ospedale San Raffaele Milano | 245 |
| Ospedale Sant'Anna Como | 41 |
| Ospedale del Ponte Varese | 317 |
| Policlinico San Pietro Ponte San Pietro | 125 |
| Ca' Granda Ospedale Maggiore Policlinico Milano | 356 |
| Presidio Ospedaliero di Vimercate | 159 |
| San Matteo di Pavia | 174 |
| Spedali Civili Brescia | 258 |
| Presidio Ospedaliero Di Sondrio | 34 |
| Ospedale Vittore Buzzi Milano | 592 |

**Comparison between 2018-19 season and other periods**

The number of hospitalized patients was 1618 in 2018-19. After a decrease (n=121) in 2021-2022, a progressive and substantial increase occurred in 2021-22 (n=1577) and 2022-23 (n=2014).

Some differences among the four periods were observed regarding the prevalence of patients hospitalized before the autumn-winter season, the prevalence of patients aged >12 months and the etiologic cause of bronchiolitis. In particular, the percentage of hospitalized subjects between July and September was higher in 2020-21 (20%, n=25; p<0.001), whereas no significant difference was observed in 2021-22 (1.6%, n=25; p=0.886) and in 2022-23 (1.9%, n=38; p=0.369) as compared to 2018-19 (1.5%, n=24). Compared to 2018-19 (5.9%, n=96), the prevalence of patients older than 12 months was higher in 2020-21 (19%, n=23; p<0.001), and similar in 2021-22 (4.9%, n=77; p=0.211) and in 2022-23 (7.0%, n=140; p=0.223). Compared to 2018-19 (56%, n=897), the prevalence of respiratory syncytial virus was lower in 2020-21 (6.6%, n=8; p<0.001), and higher in 2021-22 (71%, n=1120; p<0.001) and in 2022-23 (63%, n=1267; p<0.001). Compared to 2018-19, no difference in the history of breastfeeding and the number of older siblings was found in 2021-22 and in 2022-23. In contrast, differences in age, sex, history of maternal COVID-19 during pregnancy, number of older siblings, body weight at birth, breastfeeding duration, presence of chronic diseases, radiologically confirmed diagnosis of pneumonia were observed during the study periods (table 1).

Compared to 2018-19 (59%, n=959), the prevalence of patients with O_2_-supplementation was similar in 2020-21 (57%, n=69; p=0.632) and higher in 2021-22 (73%, n=1157; p<0.001) and in 2022-23 (76%, n=1527; p<0.001). Compared to 2018-19 (median 0, IQR 0-4, days), the length of O_2_-supplementation was similar in 2020-21 (median 1, IQR 0-3, days; p=0.878) and higher in 2021-22 (median 2, IQR 0-5, days; p<0.001) and 2022-23 (median 2, IQR 0-5, days; p<0.001). Compared to 2018-19 (29%, n=470), the prevalence of patients receiving non-invasive ventilation support was similar in 2020-21 (35%, n=41; p=0.214) and higher in 2021-22 (46%, n=726; p<0.001) and in 2022-23 (52%, n=1055; p<0.001). Compared to 2018-19 (11%, n=178), the prevalence of patients admitted to intensive care was similar in 2020-21 (11%, n=13; p=0.999) and higher in 2021-22 (15%, n=239; p<0.001) and in 2022-23 (16%, n=320; p<0.001). The length of intensive care stay was similar in 2020-21 (median 3, IQR 2-6, days; p=0.101), in 2021-22 (median 5, IQR 3-7, days; p =0.006) and in 2022-23 (median 5, IQR 4-8, days, p=0.417) as compared to 2018-19 (median 5, IQR 3-7; days). Compared to 2018-19 (80%, n=143), the prevalence of patients admitted to intensive care for ≥3 days was similar in 2020-21 (62%, n=8; p=0.151) and 2021-22 (80%, n=92; p=0.151), and higher in 2022-23 (89%, n=285; p=0.04).

Compared to 2018-19 (median 6, IQR 4-8; days), the length of whole hospitalization was lower in 2020-21 (median 5, IQR 4-6, days; p<0.001) and higher in 2021-22 (median 6, IQR 4-8, day; p=0.024) and 2022-23 (median 6, IQR 4-8, days; p<0.001). Compared to 2018-19 (84%, n=1352), the prevalence of patients requiring ≥4 days of hospitalization was similar in 2020-21 (75%, n=91; p=0.051) and 2021-22 (85%, n=1343; p=0.224), and higher in 2022-23 (87%, n=1758; p=0.011).

**Table S2**. Results of the mixed effect regression models limited to infants 12 months of age or less (n=4914): oxygen supplementation, non-invasive ventilation support, invasive ventilation support, intensive care admission were the dependent variables. Study periods (reference 2018-19) were the predictive variables. Models were adjusted for age, sex, gestational age at birth, underlying chronic disease (yes vs no), history of breastfeeding, number of older siblings (no older sibling vs one or more older siblings) and testing positive for respiratory syncytial virus.

| **Outcome** | **Study periods** | **Odds ratio** | **Lower 95% confidence interval** | **Upper 95% confidence interval** | **p** |
| --- | --- | --- | --- | --- | --- |
| Need for oxygen supplementation | 2020-21 | 0.339 | 0.390 | 1.202 | 0.164 |
|  | 2021-22 | 1.240 | 1.289 | 2.063 | <0.001 |
|  | 2022-23 | 1.662 | 1.685 | 2.915 | <0.001 |
| Need for non-invasive ventilation support | 2020-21 | 1.196 | 0.578 | 2.472 | 0.630 |
|  | 2021-22 | 2.137 | 1.635 | 2.794 | <0.001 |
|  | 2022-23 | 3.580 | 2.667 | 4.806 | <0.001 |
| Need for invasive ventilation support | 2020-21 | 2.175 | 0.241 | 19.618 | 0.489 |
|  | 2021-22 | 0.592 | 0.225 | 1.555 | 0.287 |
|  | 2022-23 | 1.514 | 0.651 | 3.523 | 0.336 |
| Need for intensive care unit admission | 2020-21 | 2.515 | 0.248 | 25.471 | 0.435 |
|  | 2021-22 | 0.432 | 0.157 | 1.187 | 0.104 |
|  | 2022-23 | 1.271 | 0.544 | 1.970 | 0.580 |

**Table S3**. Results of the mixed effect regression models limited to infants 12 months of age or less (n=4914): length of intensive care unit stay, oxygen supplementation, and overall hospitalization were the dependent variables. Study periods (reference 2018-19) were the predictive variables. Models were adjusted for age, sex, gestational age at birth, underlying chronic disease (yes vs no), history of breastfeeding, number of older siblings (no older sibling vs one or more older siblings) and testing positive for respiratory syncytial virus.

| **Outcome** | **Study periods** | **ß** | **Lower 95% confidence interval** | **Upper 95% confidence interval** | **p** |
| --- | --- | --- | --- | --- | --- |
| Length of intensive care unit stay | 2020-21 | 0.107 | -6.025 | 0.840 | 0.774 |
|  | 2021-22 | 0.036 | -2.137 | 0.309 | 0.796 |
|  | 2022-23 | 0.145 | -0.151 | 0.440 | 0.337 |
| Length of oxygen supplementation | 2020-21 | -0.909 | -1.172 | -0.045 | 0.039 |
|  | 2021-22 | 0.768 | 0.447 | 1.089 | <0.001 |
|  | 2022-23 | 1.160 | 0.814 | 1.506 | <0.001 |
| Whole duration of hospital stay | 2020-21 | -0.826 | -1.846 | 0.194 | 0.113 |
|  | 2021-22 | -0.057 | -0.442 | 0.328 | 0.773 |
|  | 2022-23 | 0.630 | 0.214 | 1.046 | 0.003 |
